# Six Months of Inspiratory Muscle Training to Lower Blood Pressure and Improve Endothelial Function in Middle-Aged and Older Adults With Above-Normal Blood Pressure and Obstructive Sleep Apnea: Protocol for the CHART Clinical Trial

**DOI:** 10.3389/fcvm.2021.760203

**Published:** 2021-11-24

**Authors:** Dallin Tavoian, Lupita E. Ramos-Barrera, Daniel H. Craighead, Douglas R. Seals, Edward J. Bedrick, Joseph S. Alpert, Saif Mashaqi, E. Fiona Bailey

**Affiliations:** ^1^Arizona Respiratory Neurophysiology Laboratory, Department of Physiology, University of Arizona, Tucson, AZ, United States; ^2^Department of Integrative Physiology, University of Colorado Boulder, Boulder, CO, United States; ^3^Department of Epidemiology and Biostatistics, Mel and Enid Zuckerman College of Public Health, University of Arizona, Tucson, AZ, United States; ^4^College of Medicine, University of Arizona, Tucson, AZ, United States; ^5^Division of Cardiology, Sarver Heart Center, College of Medicine, University of Arizona, Tucson, AZ, United States; ^6^Division of Pulmonary, Allergy, Critical Care and Sleep Medicine, Banner – University Medical Center, Tucson, AZ, United States; ^7^Sleep Disorders Center, Banner – University Medical Center, Tucson, AZ, United States

**Keywords:** obstructive sleep apnea, hypertension, inspiratory, exercise, older adults, blood pressure, endothelial, vascular

## Abstract

**Background:** Cardiovascular disease is a major global health concern and prevalence is high in adults with obstructive sleep apnea (OSA). Lowering blood pressure (BP) can greatly reduce cardiovascular disease risk and physical activity is routinely prescribed to achieve this goal. Unfortunately, many adults with OSA suffer from fatigue, daytime sleepiness, and exercise intolerance—due to poor sleep quality and nocturnal hypoxemia—and have difficulty initiating and maintaining an exercise program. High-resistance inspiratory muscle strength training (IMST) is a simple, time-efficient breathing exercise consistently reported to reduce BP in small, selective groups of both healthy and at-risk adults. Herein we present the study protocol for a randomized clinical trial to determine the long-term efficacy of IMST performed regularly for 24 weeks in middle-aged and older adults with OSA. The primary outcome is casual systolic BP. Secondary outcomes are 24-h systolic BP and circulating plasma norepinephrine concentration. Other outcomes include vascular endothelial function (endothelial-dependent and -independent dilation), aortic stiffness, casual and 24-h diastolic BP, and the influence of circulating factors on endothelial cell nitric oxide and reactive oxygen species production. Overall, this trial will establish efficacy of high-resistance IMST for lowering BP and improving cardiovascular health in middle-aged and older adults with OSA.

**Methods:** This is a single-site, double-blind, randomized clinical trial. A minimum of 92 and maximum of 122 male and female adults aged 50–80 years with OSA and above-normal BP will be enrolled. After completion of baseline assessments, subjects will be randomized in a 1:1 ratio to participate in either high-resistance or sham (low-resistance) control IMST, performed at home, 5 min/day, 5 days/week, for 24 weeks. Repeat assessments will be taken after the 24-week intervention, and after 4 and 12 weeks of free living.

**Discussion:** This study is designed to assess the effects of 24 weeks of IMST on BP and vascular function. The results will characterize the extent to which IMST can reduce BP when performed over longer periods (i.e., 6 months) than have been assessed previously. Additionally, this study will help to determine underlying mechanisms driving IMST-induced BP reductions that have been reported previously.

**Clinical Trial Registration:** This trial is registered with ClinicalTrials.gov (Registration Number: NCT04932447; Date of registration June 21, 2021).

## Introduction

### Background and Rationale

Cardiovascular disease (CVD) is the leading cause of death in the US and worldwide (1, 2). Obstructive sleep apnea (OSA) is a chronic sleep-related breathing disorder, characterized by repeated partial (hypopnea) or complete (apnea) collapse of the pharyngeal airway, with higher-than-average rates of CVD (3). OSA prevalence has risen sharply over recent decades (4), with current estimates indicating one billion adults (ages 30–69 years) are affected worldwide and prevalence rates in the US nearing 48% (5).

From a physiological perspective, airflow limitation and obstructions often result in chronic intermittent hypoxemia, arousal from sleep, and bursts of sympathetic activity that provoke surges in blood pressure (BP) and heart rate (6–9). In turn, chronic intermittent hypoxemia promotes reactive oxygen species (ROS) overproduction and contributes to chronic systemic inflammation (10, 11). The health implications are especially profound for older adults (65+ years of age) regardless of OSA status, as over 80% of all cardiovascular deaths occur in this age group (12). Age-related stiffening of the large elastic arteries (i.e., aortic and carotid) (13–16) and vascular endothelial dysfunction compound the problem.

The primary therapy for OSA is continuous positive airway pressure (CPAP), which delivers a steady stream of pressurized air *via* a mask that stents the airway, preserving airflow and oxygenation. CPAP is highly efficacious among adherent users (≥4 h/night, 5 nights a week) as it normalizes sleep architecture (17, 18), reduces sympathetic nervous system activity (19, 20) and lowers systolic BP (SBP) (2–6 mmHg) (21, 22). Despite enhanced usability, CPAP adherence rates remain low (30–60%) (23), with ~50% of patients discontinuing use within the first year (24). Low adherence rates limit the therapeutic impact of CPAP for ongoing CVD and/or CVD development (25–27).

Regular aerobic exercise has important physiologic and clinical benefits in adults with above-normal BP and/or OSA, including reductions in BP (28), OSA severity (29), and risk for adverse cardiovascular events (30, 31). Unfortunately, the salient features of OSA, including obesity (BMI >30 kg/m^2^), excessive daytime sleepiness (32), and lethargy (33–37), render many adults with this disorder unwilling or unable to exercise, or cause them to discontinue exercise before achieving health gains (38, 39). Time availability is a major barrier to exercise adherence (39, 40) and the need to establish the efficacy of novel forms of exercise that are time-efficient, well-tolerated, and effective has never been greater (41, 42).

**Figure 1:**
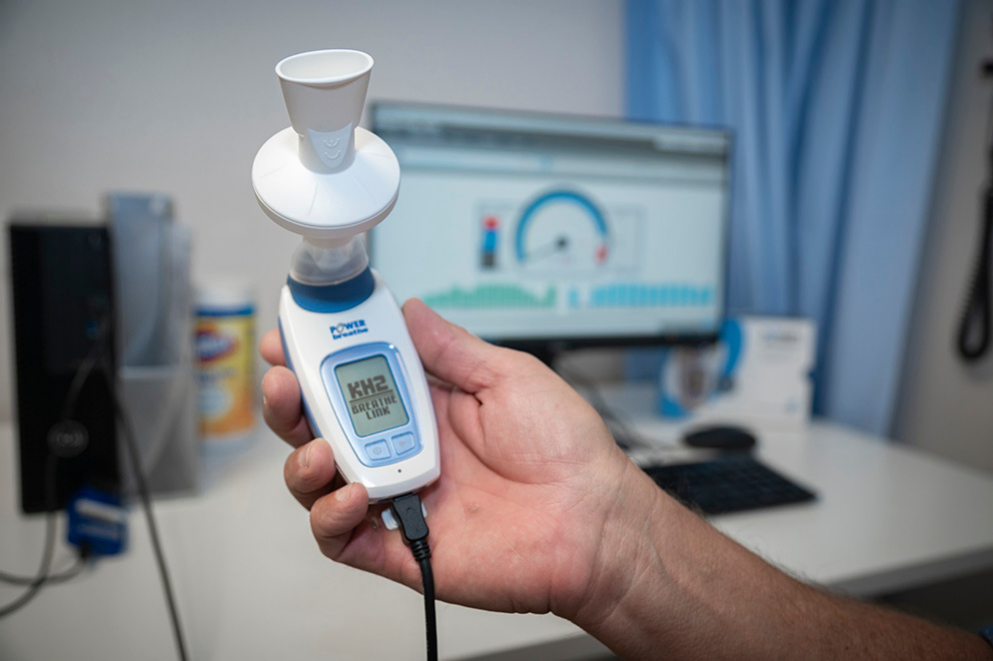
Handheld POWERbreathe training device.

Among the exercise modalities that offer improved adherence, shorter duration of effort [relative to current guidelines (43)], and BP-lowering effects, is a respiratory training protocol known as inspiratory muscle strength training, or IMST (42). IMST is performed on a portable hand-held device (Figure 1) and entails repeated inspiratory efforts against a resistance. IMST is distinct from more traditional forms of aerobic exercise in that it is performed (a) in stationary sitting or standing, (b) at slower breathing rates (~10–12 breaths/min), and (c) requires large inspiratory (i.e., negative) pressures (44, 45) that exceed those generated in tidal breathing (46), deep breathing (45), or high-intensity aerobic exercise (i.e., 95% VO_2_max) (47, 48). Another key distinction is a very abbreviated training format which, for high resistance IMST, comprises just 30 breaths (5 min/day), or a total training time of ~30 min per week.

Over the short-term (i.e., 6 weeks), high-resistance IMST has yielded improvements in SBP, sympathetic nervous system activity, and endothelial function in small groups of healthy and at-risk populations (45, 49–52), including middle aged and older (MA/O) adults with OSA. However, the results for OSA adults are preliminary and require confirmation in a larger group of patients. Furthermore, guidelines issued by the American College of Cardiology and the American Heart Association (ACC/AHA) indicate that non-pharmacological therapies designed to reduce BP should be assessed for efficacy within 3–6 months of treatment initiation (28), and the efficacy of high-resistance IMST administered over the longer-term has yet to be assessed. By extension, it is not known how long IMST-induced BP reductions persist after training cessation. Lastly, in view of evidence that high-resistance IMST improves endothelial function in otherwise healthy MA/O adults with above-normal BP (52), the potential for IMST to induce similar benefits in MA/O adults with OSA warrants assessment.

The CHART (Cardiovascular Health, Apnea, and Respiratory Training) Study is a Phase II clinical trial that seeks to establish the benefits of 24 weeks IMST on: (1) casual (resting) and 24-h (ambulatory) SBP, and (2) to determine if the reductions in SBP are sustained following cessation of IMST. In addition, we seek to understand the underlying mechanisms that drive IMST-related BP reductions. Specifically, we will assess the effects of IMST on: (1) markers of sympathetic nervous system activity and other potential vasoconstrictor factors, arterial stiffness, nitric-oxide (NO)-mediated endothelial function (endothelial-dependent dilation), smooth muscle sensitivity to NO (endothelial-independent dilation), and systemic oxidative stress and inflammation; and (2) the influence of changes in circulating factors on the release of NO and ROS from human umbilical vein endothelial cells *ex vivo*, using a novel cell-culture model. To achieve these objectives, we will assess the effects of high- vs. low-resistance IMST performed at home 5 min/day, 5 days/week, for 24 weeks in adults 50–80 years of age with OSA (apnea-hypopnea index ≥15) and above-normal SBP (≥120 mmHg). Further, we will reassess these measures after 4 and 12 weeks of free-living following cessation of the 24-week training intervention.

## Methods

### Study Design

**Figure 2:**
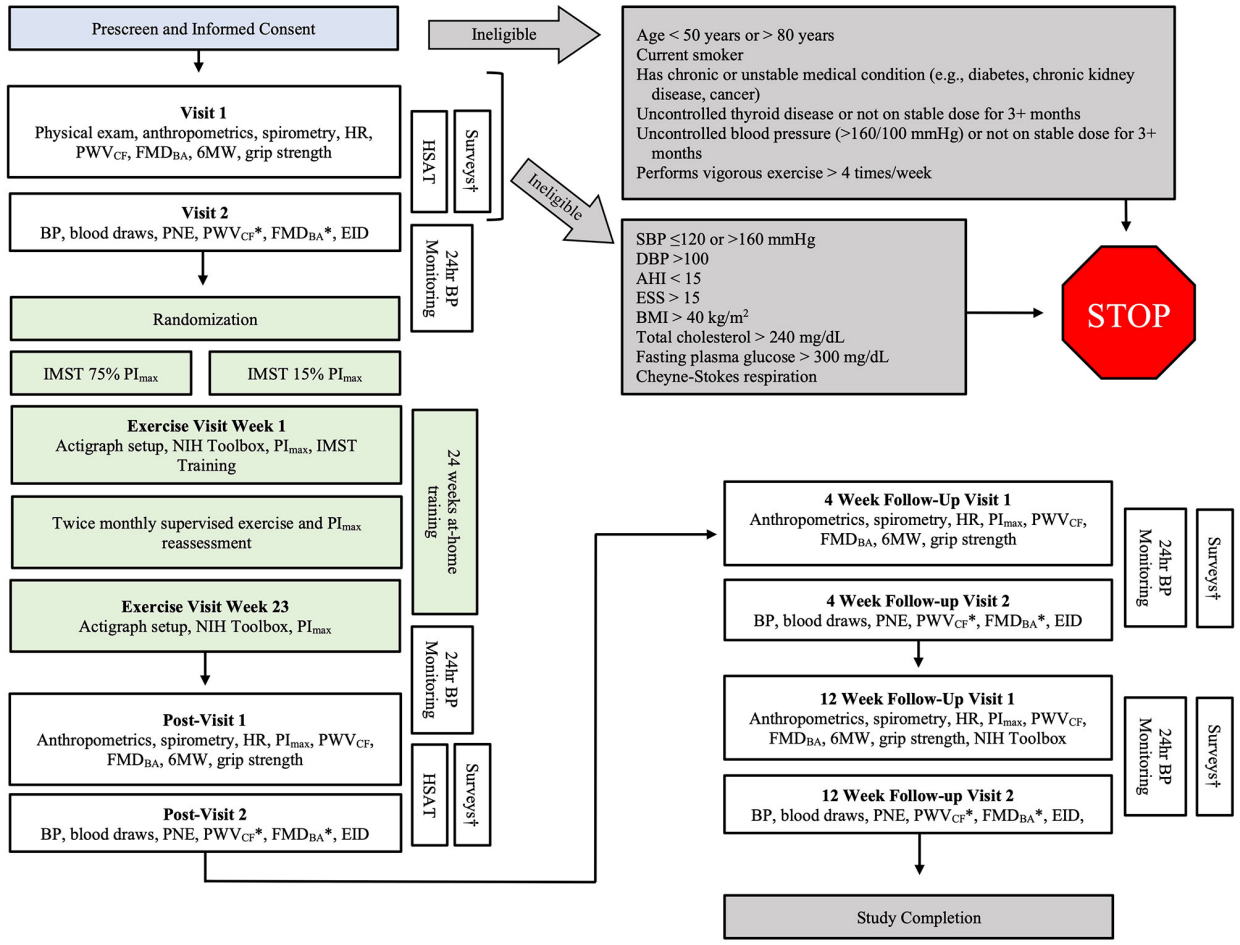
Detailed overview of the CHART study protocol. 6MW, six-minute walk; AHI, apnea hypopnea index; BMI, body mass index; BP, blood pressure; CHAMPS, Community Healthy Activities Model Program for Seniors physical activity questionnaire; ESS, Epworth Sleepiness Scale; FMD_BA_, brachial artery flow-mediated dilation; HR, heart rate; IMST, inspiratory muscle strength training; PI_max_, maximal inspiratory pressure; PNE, plasma norepinephrine; PSQI, Pittsburgh Sleep Quality Index; PWV_CF_, carotid-femoral pulse wave velocity; SBP, systolic blood pressure; *Indicates PWV_CF_ and FMD_BA_ will be performed with saline and Vitamin C infusion. ^†^The following surveys will be completed at home: CHAMPS, ESS, PSQI.

The CHART Study is a Phase II randomized, double-blind, sham-controlled, mechanistic clinical trial comparing high- and low-resistance IMST in 122 MA/O adults (50–80 years) with OSA. The study is a repeated measures design with assessments at four time points: baseline, post intervention, and at follow-up 4- and 12-weeks post-intervention (Figure 2).

### Study Population

Men and women 50–80 years of age with OSA and above-normal BP from all ethnic backgrounds will be eligible to participate. Subjects will be screened to determine whether they meet all the inclusion criteria and have none of the exclusion criteria (Table 1).

**Table 1:** Inclusion and exclusion criteria.

**Inclusion**	**Exclusion**
• Age 50–80 years • Ability to understand study procedures and to comply with them for the entire length of the study • Ability to provide informed consent; • Willing to accept random assignment to condition • AHI ≥15 • Individuals with who are unwilling or unable to adhere to CPAP • Individuals who are adherent to CPAP therapy (i.e., 4 h/night on 70%/nights over 30 days in the first 3 months of initial usage) • Individuals who are adherent to mandibular advancement device each night • Above-normal SBP (i.e., SBP ≥120) • BMI ≤ 40 kg/m^2^ • Weight stable in the prior 3 months (<3.0 kg weight change) and willing to remain weight stable throughout the study • No change in anti-hypertensive medications or other medications (prescription or dosing) in the prior 3 months and willingness to maintain current medication regimen throughout the study • Absence of unstable clinical disease as determined by medical history, physical examination, and blood chemistries • Total cholesterol <240 mg/dL • Fasting plasma glucose <300 mg/dL	• Age <50 or >80 years • AHI <15 • BMI > 40 kg/m^2^ • Individuals with central or mixed sleep disordered breathing • Severe hypoxemia (<80% for >10% of recording time) during sleep • ESS >15 • SBP ≤ 120 or >160 • DBP >100 • Current smoker • Chronic overt and poorly controlled medical condition (e.g., diabetes, chronic kidney disease, cancer, congestive heart failure) • Cheyne-Stokes Respiration • Alcohol or illegal drug dependence or abuse • Uncontrolled thyroid disease or change in thyroid medication within previous 3 months • Regular/vigorous aerobic exercise (> 4 bouts/week, >30 min/bout at high workload >6 METS)

*AHI, apnea hypopnea index; BMI, body mass index; CPAP, continuous positive airway pressure; DBP, diastolic blood pressure; ESS, Epworth Sleepiness Scale; METS, metabolic equivalents; SBP, systolic blood pressure*.

### Outcomes

#### Primary Outcome

##### Casual Systolic Blood Pressure

Casual SBP will be assessed in accordance with ACC/AHA guidelines (28) between 7 and 10 a.m. and after overnight fasting. Subjects will be seated quietly with their back supported, feet flat on the floor, and arms supported on a table at heart level with the palms facing up. SBP will be measured with an automated oscillometric sphygmomanometer (SunTech CT40, SunTech Medical), validated according to standards set by the British Hypertension Society, International Protocol and Association for the Advancement of Medical Instrumentation (53), and calibrated regularly by the manufacturer. Measurements will be performed in triplicate over the brachial artery of the non-dominant arm after at least 5 min of quiet rest and with 1 min of recovery between measures. SBP will be defined as the average of the three pressures, and will be recorded at baseline, weekly throughout the intervention, after the 24-week training period, and at 4- and 12-weeks post-intervention.

#### Secondary Outcomes

##### Twenty-Four-Hour Ambulatory Systolic Blood Pressure

Twenty-four-hour SBP will be assessed with 24-h ambulatory non-invasive BP monitoring. Given the propensity for sleep disturbance/arousal reaction to contribute to elevations of BP, assessments will be continuous, beat-to-beat over a 24-h period with the and European Association of Hypertension validated cuffless monitor (SOMNOtouch, SOMNOmedics Germany) (54). Participants will be given a diary to record any physical or psychological events that occur during the 24-h period that may influence BP. SBP will be averaged over the entire 24-h period at baseline, after the 24-week training period, and at 4- and 12-weeks post-intervention.

##### Daytime and Nighttime Ambulatory Systolic Blood Pressure

Ambulatory SBP data will be broken into daytime (16 h) and nighttime (8 h) segments according to subjects' individual sleep-wake cycles. Mean SBP (mmHg) will be calculated for daytime and nighttime periods to evaluate circadian SBP profile at baseline, after the 24-week training period, and at 4- and 12-weeks post-intervention.

##### Circulating Plasma Norepinephrine

Concentration of circulating PNE (pg/mL) will be determined from venous blood samples acquired following 20 min of supine rest in a quiet, temperature-controlled room. PNE will be quantified *via* high-performance liquid chromatography (55) from blood collected at baseline, after the 24-week training period, and at 4- and 12-weeks post-intervention.

#### Other Outcomes

##### Endothelial Dependent Dilation

EDD will be assessed *via* brachial artery flow-mediated dilation (FMD_BA_) using high-resolution ultrasonography (Canon Xario 200G) (52, 56), the gold-standard *in vivo* measure of conduit artery vascular endothelial function, that is primarily mediated by NO. FMD_BA_ will be assessed by measuring brachial artery diameter and blood velocity at baseline and for 3 min following reactive hyperemia induced by 5 min of forearm blood flow occlusion with a cuff placed on the upper forearm and inflated at least 50 mmHg above SBP.

Data will be expressed as both percent and absolute (mm) change in arterial diameter from baseline (pre-cuff inflation diameter). Shear area-under-the-curve up to peak diameter will be calculated, and if group- or timepoint-differences exist, FMD_BA_ will be adjusted accordingly. Brachial artery diameter and blood velocity will be analyzed offline with commercially available software (Brachial Analyzer, Medical Imaging Applications LLC, Coralville, IA, USA). Peak arterial diameter will be compared to baseline diameter to calculate absolute change (mm) and percent change in diameter in response to the forearm hyperemic stimulus at baseline, after the 24-week training period, and at 4- and 12-weeks post-intervention.

To determine tonic oxidative stress-mediated suppression of endothelial function, FMD_BA_ will be repeated after intravenous infusion of supratherapeutic concentrations of the potent ROS-scavenging antioxidant, Vitamin C, and isovolumic saline infusion, as described previously (57, 58). A bolus of 0.06 g Vitamin C/kg fat-free mass dissolved in 100 mL of saline will be infused by IV at a rate of 5 mL/min for 20 min, followed immediately by a drip infusion of 0.02 g/kg fat-free mass dissolved in 30 mL saline infusion at a rate of 0.5 mL/min. FMD_BA_ will be performed after the bolus dose, during the drip infusion. FMD_BA_ with infusions will be performed at baseline, after the 24-week training period, and at 4- and 12-weeks post-intervention.

##### Endothelial Independent Dilation

EID will be assessed in response to sublingual nitroglycerin to assess smooth muscle sensitivity to NO. EID will be determined by measuring brachial artery dilation for 8 min after sublingual nitroglycerin administration (0.4 mg) using high-resolution ultrasonography, as described above. Absolute change and percent change in arterial diameter will be used for analysis. EID will be performed at baseline, after the 24-week training period, and at 4- and 12-weeks post-intervention.

##### Arterial Stiffness

Arterial stiffness will be assessed *via* carotid-femoral pulse wave velocity (PWV_CF_) using a transcutaneous tonometer (SPT-301 ADInstruments, Colorado Springs, CO) placed sequentially in position at the carotid and femoral arteries, with simultaneous electrocardiograph (ECG) recording (59). PWV_CF_ will be calculated as mean distance/transit time. Transit time will be determined as the time between the ECG R-wave and carotid pulse subtracted from the time between the ECG R-wave and femoral pulse. Thirty consecutive pulse waves from each arterial site will be analyzed and averaged. PWV_CF_ will be calculated from difference in carotid-sternal and femoral-sternal distance (m) divided by difference in transit time (s). PWV_CF_ will be assessed at baseline, after the 24-week training period, and at 4- and 12-weeks post-intervention.

##### Influence of Circulating Factors on Nitric Oxide and Reactive Oxygen Species Production

Using blood drawn from experimental groups we will determine if IMST improves endothelial function *via* changes in circulating factors that increase endothelial cell NO production and/or reduce endothelial ROS production in cultured human umbilical vein endothelial cells (HUVECs) (American Type Culture Collection). We will expose HUVECs *ex vivo*, to media conditioned with serum drawn from participants in each group as described previously (52). After 2–4 passages, HUVECs will be plated in 96-well culture plates and incubated for 24 h at 37°C and 5% CO_2_ in basal media supplemented with 10% subject serum obtain before and after 24 weeks of low-resistance or high-resistance IMST. After incubation, cells will be co-incubated with fluorescent probes DAR-4M AM to detect NO production, and CellROX Deep Red to detect ROS production. Cells imaged with DAR-4M AM will be imaged 5 min after the addition of 200 μmol/L of acetylcholine to the cell culture media to stimulate NO production. Analysis will be done with Image J (National Institutes of Health). Data will be expressed as a fold change in NO/ROS production relative to the baseline timepoint.

##### Casual Diastolic Blood Pressure

Casual (resting) measures of DBP will be measured according to ACC/AHA guidelines (28). Measures will be made in triplicate over the brachial artery of the non-dominant arm after 5 min of quiet rest, with 1 min of recovery between each measure. Subjects will be seated quietly with their back supported, feet flat on the floor, and arm at heart level. DBP will be measured with an automated oscillometric sphygmomanometer (SunTech CT40, SunTech Medical), validated according to British Hypertension Society, International Protocol, and Association of Medical Instrumentation standards. Mean DBP will be calculated as the average of the three pressures, and will be recorded at baseline, weekly throughout the intervention, post intervention, and at 4- and 12-weeks post-intervention.

##### Twenty-Four-Hour Ambulatory Diastolic Blood Pressure

Twenty-four-hour DBP will be assessed with 24-h ambulatory non-invasive BP monitoring, as described above. Ambulatory DBP will be averaged over the entire 24-h period at baseline, post intervention, and at follow-up 4- and 12-weeks after cessation of the intervention.

##### Daytime and Nighttime Ambulatory Diastolic Blood Pressure

Ambulatory DBP data will be broken into daytime (16 h) and nighttime (8 h) segments according to subjects' individual sleep-wake cycles. Mean DBP (mmHg) will be calculated for the daytime and nighttime periods to evaluate circadian DBP profile at baseline, after the 24-week training period, and at 4- and 12-weeks post-intervention.

##### Six-Minute Walk Distance

A six-minute walk test will be performed in a 30-m hallway with cones to mark the ends of the track. The subject will be instructed to walk as quickly as they can, without running, for 6 min. They will start at the first cone and walk down the hallway, rounding the second cone, and returning up the hallway toward the first cone. They will continue to walk up and down the hallway, completing as many laps as possible in 6 min. Subjects will be told how much time is remaining every minute of the test, and with 30 and 10 s remaining, and encouraged to walk as quickly as possible each time. Distance covered in 6 min will be recorded at baseline, after the 24-week training period, and at 4- and 12-weeks post-intervention.

##### NIH Toolbox—Cognitive Domain

Subjects will complete the 7 cognitive domain assessments from the NIH Toolbox for the Assessment of Neurological and Behavioral Function (60). These tests include: (1) flanker inhibitory control and attention test; (2) dimensional change card sort test; (3) list sorting working memory test; (4) picture sequence memory test; (5) oral reading recognition test; (6) picture vocabulary test; and (7) pattern comparison processing speed test. All tests will be performed on an iPad (Apple Inc, Cupertino, CA, USA) with an investigator presenting task instructions and monitoring compliance, as recommended by the American Academy of Neurology (60). Scores will be recorded at baseline after the 24-week training period, and at 12-weeks post-intervention.

##### Maximal Inspiratory Pressure

Maximal inspiratory pressure (PI_max_) will be assessed by having subjects generate 3 maximal inspiratory efforts against near-infinite resistance measured at the mouth with a POWERbreathe™ KH2 trainer (POWERbreathe™ KH2, POWERbreathe International Ltd., Warwickshire, ENG). The KH2 includes computer software (Breathe-Link Medic, POWERbreathe International Ltd., Warwickshire, ENG) that gives live feedback and can assess PI_max_ and peak inspiratory flow. If there is >10% difference between efforts, subjects will perform up to 3 more efforts to ensure PI_max_ is achieved. The average of the 3 greatest pressures will be used for analysis, and will be assessed at the first exercise session, after the 24-week training period, and at 4- and 12-weeks post-intervention.

##### Resting Heart Rate

Resting heart rate will be measured simultaneously with blood pressure using the same automated oscillometric sphygmomanometer as is used for blood pressure assessments (SunTech CT40, SunTech Medical). As with blood pressure, heart rate will be defined as the average of the three readings at baseline, and will be performed at baseline, after the 24-week training period, and at 4- and 12-weeks post-intervention.

##### Home Sleep Apnea Testing

Severity of obstructive sleep apnea (i.e., AHI) will be assessed with a Nox T3s Home Sleep Apnea Testing diagnostic device (Nox T3s, Nox Medical, Reykjavik, IS), a Type III polygraph with actigraphy. Subjects will be instructed on the use and fitting of the device. They will be instructed to turn on the device immediately prior to going to bed and to turn it off upon waking. Subjects with an AHI < 15 will be excluded from the study. Outcomes include total sleep time, apneas/hour, hypopneas/hour, average O_2_ saturation (SpO_2_), minimum SpO_2_, duration of SpO_2_ < 90%, and duration of SpO_2_ < 85%. Home sleep apnea testing will be performed at baseline and after the 24-week training period, but not at 4- and 12-weeks post-intervention.

##### Grip Strength

Maximal grip strength will be obtained with a Jamar hydraulic grip strength dynamometer (Performance Health, LLC, Akron, OH USA). Handle position of the dynamometer will be standardized at position II (61). The subject will be seated with the shoulder in neutral position and the elbow supported and flexed to 90° with the forearm and wrist in neutral position. The subject will squeeze the dynamometer handle as hard as possible for 3 s and maximal force will be recorded in kg. The subject will perform three trials with each hand, and 15 s rest will be allowed between trials. Peak force of each hand will be used for analysis. Grip strength will be performed at baseline, after the 24-week training period, and at 4- and 12-weeks post-intervention.

##### Muscle Sympathetic Nerve Activity

Muscle sympathetic nerve activity (MSNA) will be recorded from the common peroneal nerve *via* tungsten microelectrode (200 μm: 25–40 mm, impedance: 5 MX) (FHC, Bowdoin, ME) inserted percutaneously immediately posterior to the fibular head. Due to the technically difficult and time-consuming nature of MSNA, it will be assessed in a subset of subjects in the CHART study (*n* = 10/group). Subjects selected to complete MSNA testing will do so at a visit separate from Visit 1 and Visit 2 at baseline, after the 24-week intervention, and at the 12-week follow-up. Subjects will rest in a chair reclined ~45° with the right knee and foot supported by positioning pillows (VersaForm, Performance Health, Warrenville, IL). Microelectrode placement will be confirmed *via* electrical stimulation (0.02 mA, 1 Hz) as described previously (62, 63). A second microelectrode will be inserted just below the skin surface ~1.0 cm from the first to serve as a reference electrode. Electrode position in sympathetic fascicles will be confirmed by pulse synchronous bursts of activity, elicitation of afferent nerve activity by mild muscle stretch, and absence of response to light stroking of the skin or with startle response to loud noises (62). The recorded signal will be amplified (gain 2 × 10^4^), bandpass filtered (500–2.0 kHz) using a pre-amplifier (NeuroAmp Ex; ADInstruments, Colorado Springs, CO), and signals will be full-wave rectified (100 ms moving window) and sampled at 10 kHz. The resulting signal will be monitored using a computer-based data acquisition and analysis system (LabChart 8.0 software, ADInstruments, Colorado Springs, CO) and loudspeaker throughout the experiment. MSNA will be recorded for 10 min, and bursts that exceed a predetermined threshold will be marked and counted. Outcomes will include bursts/minute, bursts/100 heart beats, and mean burst area/minute.

### Sample Size Calculation and Power

A minimum of 92 and maximum of 122 participants will be enrolled and randomized in this study (*n* = 46–61 per group). As OSA prevalence is 2-fold greater in males than females over the age of 50 (64–66), we expect to enroll males at a 2:1 ratio to females. However, we will not restrict enrollment based on sex.

In our preliminary study (49), the effect sizes (ESs) for SBP, PNE, and 24-h SBP were 2.50, 1.25, and 0.63. The within individual standard deviation (SD) was set to be the larger of the SDs for the two groups, although differences in SDs across groups were not substantial. If 45 participants per arm complete the main study, we will have over 80% power for any outcome with an ES > 0.63. Sex-specific comparisons of mean change scores between IMST groups can detect an ES > 1.19, provided at least 30 females complete the study (i.e., 75% of 1/3 of the initial sample size of 122), with at least 15 in each arm. This calculation includes a Bonferroni correction of the significance level—that is, the sex-specific comparisons are conducted at the 2.5% significance level.

## Treatment of Subjects

### Study Location and Timeline

All baseline, training, post-assessment and follow up measures will be conducted at the University of Arizona's Clinical and Translational Research Services (CATS) facility. The enrollment period for an individual participant will be ~42 weeks. The timeline for the entire study (i.e., recruitment of first subject to study completion for final subject) will be ~54 months.

Subjects who have been screened will have 6 months from the prescreening date to commence the study. If after 6 months they have not attended the first baseline session, they will be required to undergo prescreening assessments again prior to proceeding to baseline assessments.

Two baseline sessions, spaced at least 24 h apart, will be completed within 14 days. Participants will commence the intervention within 14 days of the second baseline assessment and perform their prescribed exercise at home, 5 days/week for 24 weeks. Post-intervention assessments will be completed within a 14-day period, at least 24 h apart and in week 25 ± 1 week. Follow-up assessments will be performed within a 14-day period, at least 24 h apart at 4 ± 1 weeks and 12 ± 1 weeks after the last post-intervention assessment (Table 2).

**Table 2:** Schedule of events.

	**Baseline**		**Exercise intervention**				**Post**		**F4 and F12**	
	**V1**	**V2**	**Wk 1**	**Wks 2–4**	**Wks 6–22**	**Wk 23**	**V1**	**V2**	**V1**	**V2**
Day (window)	−14–0		1–7	8–28	29–154	147-154	168–182		F4: 203–217 F12: 259–273	
**Screening/baseline**										
Informed consent	X									
Demographics	X									
Medical Hx/physical exam	X									
Inclusion/exclusion	X									
Height/weight	X						X		X	
Vitals	X	X	X	X	X	X	X	X	X	X
Neck circumference	X						X		X	
**Cognitive assessments**										
NIH Toolbox			X			X			
**Surveys**										
CHAMPS	X						X		X
PSQI	X						X		X
ESS	X						X		X
**Exercise sessions**										
Daily exercise			X	X	X	X				
Once-weekly supervised			X	X						
Twice-monthly supervised					X	X				
Weekly text/email			X	X	X	X				
**At-home testing**										
24-h BP		X				X			X
HSAT	X						X	
Actigraphy			X			X		
Sleep/exercise diary			X	X	X	X		
**Functional assessments**										
Casual BP		X						X		X
Spirometry	X						X		X
PI_max_			X	X	X	X	X		X
6MW	X						X		X
Grip strength	X						X		X
**Blood draws**										
PNE		X						X		X
Blood chemistries		X						X		X
**Arterial assessments**								
PWV_CF_	X						X		X
PWV_CF_ with NS		X						X		X
FMD_BA_	X						X		X
FMD_BA_ with NS		X						X		X
FMD_BA_ with vitamin C		X						X		X
EID with SL NTG		X						X		X
**Randomization**		X								

*6MW, six-minute walk; CHAMPS, Community Health Activities Model Program for Seniors physical activity questionnaire; BP, blood pressure; EID, endothelium-independent dilation; ESS, Epworth Sleepiness Scale; F4, first follow-up at 4 weeks; F12, second follow-up at 12 weeks; FMD_BA_, brachial artery flow-mediated dilation; HSAT, home sleep apnea testing; Hx, history; NS, normal saline infusion; NTG, nitroglycerin; PI_max_, maximal inspiratory pressure; PNE, plasma norepinephrine; PSQI, Pittsburgh Sleep Quality Index; PWV_CF_, carotid-femoral pulse wave velocity; SL, sublingual; V, visit. ^†^Surveys completed at home: CHAMPS, PSQI, ESS*.

### Baseline Assessments

Eligible participants who are enrolled into the study will undergo the Outcome Assessments and Internal Control Assessments, found in Table 3, within a period of 14 days. Subjects will be asked to maintain their behavior throughout the study, and we will account for unreported changes with the Internal Control Assessments. Protocols for these assessments can be found in the Supplementary Material.

**Table 3:** Outcome assessments and internal controls.

**Outcome assessments**	**Internal controls**
• Casual BP • 24-h BP monitoring • Blood draw • EDD *via* FMD_BA_ (with and without systemic saline and vitamin C infusion) • EID *via* sublingual nitroglycerin • Arterial stiffness *via* PWV_CF_ • 6MW • NIH Toolbox—cognitive domain • PI_max_ • Resting heart rate • Home sleep apnea testing • Grip strength • MSNA (subset of participants)	• Anthropometrics • Surveys • PSQI • ESS • CHAMPS • Actigraphy • Spirometry • Sleep and physical activity diaries

*Outcome Assessments include primary, secondary, and other outcomes of interest in the CHART study. Internal Controls will be completed to determine consistency of behavior throughout the study and to account for unreported changes. See Supplementary Material for protocols related to Internal Controls. 6MW, six-minute walk distance; BP, blood pressure; CHAMPS, Community Healthy Activities Model Program for Seniors physical activity questionnaire; EDD, endothelial-dependent dilation; EID, endothelial independent dilation; ESS, Epworth Sleepiness Scale; FMD_BA_, brachial artery flow-mediated dilation; PI_max_, maximal inspiratory pressure; PSQI, Pittsburgh Sleep Quality Index; PWV_CF_, carotid-femoral pulse wave velocity*.

### Intervention

Following baseline assessments, participants will begin the training intervention. All training will be performed on the POWERbreathe™ K3 trainer (POWERbreathe™ K3, POWERbreathe International Ltd., Warwickshire, ENG), a handheld pressure-threshold device that can: (1) be pre-programmed to provide the appropriate resistance to inspiration, and (2) record data from all training sessions. Each participant will be provided their own device on which to perform their prescribed exercise at home, 5 days/week for 24 weeks, and will receive verbal and written instruction on the training protocol and K3 operation from the unblinded Research Technician.

The Research Technician will supervise the subject's first training session to ensure proper technique and comprehension. In training weeks 1–3, participants will perform 5 training sessions per week; one training session will be supervised by the Research Technician in the CATS facility, and 4 training sessions will be performed unsupervised at home. In training weeks 4–24, participants will perform training sessions unsupervised at home returning to the CATS facility for supervised training sessions twice monthly. At each visit, the unblinded Research Technician will determine the participant's casual BP, collect sleep and activity logs, transfer saved training data from the K3 device, and record any changes in medications, diet, exercise, and/or health status since the previous visit. The participant will complete 3 PI_max_ efforts to establish the following weeks' training level. The new resistance will be set on the K3 using the *Manual* function to ensure that the target resistance remains the same until the next in-person visit. The participant then will perform one supervised training session at this new training level. Subjects will be contacted weekly *via* phone or email to encourage participants and aid in retention and adherence.

Subjects assigned to high-resistance IMST will train against a resistance set at 75% of their PI_max_. Subjects randomly assigned to low-resistance IMST will train against a resistance set at 15% of PI_max_. Subjects in both groups will perform 5 sets of 6 breaths, with 1 min rest between sets, for a total of 30 breaths per day (49). All exercise sessions will be recorded in the device memory card and participants will be required to complete a weekly training log.

### Post Assessments

To determine the chronic effects of IMST and avoid confounding by acute effects of the intervention, two post-intervention assessments will be completed within a 14-day period, at least 24 h apart beginning in week 25 ± 1 week. Participants will continue to perform daily training (except for the days they will be performing post-intervention assessments) until the post-intervention assessments are completed. Post-assessment measurements will encompass all measures obtained at baseline and will be made under the same experimental conditions, at the same time of day, in the same order and with the same techniques as for baseline testing (Table 2).

### Follow-Up Evaluations

Following completion of the post-assessments, participants will return the K3 training device and begin a period of free-living. During this period, subjects will not complete any interventions and will be asked to refrain from making any significant lifestyle changes. Subjects will be contacted weekly *via* phone or email. Primary, secondary, and other outcomes measures will be reevaluated in all participants during follow-up testing after 4 and 12 weeks of free-living. All measurements will be made under the same experimental conditions, in the same order and with the same techniques as for baseline testing, with the exception of home sleep apnea testing which will not be performed at follow-up visits (Table 2).

## Procedures

### Recruitment, Eligibility, and Consent

Recruitment will be *via* newspaper and newsletter advertisement to the general population, University of Arizona (UA) press releases, Facebook, and public presentations to local community groups in the Tucson/Oro Valley/Green Valley region. Once a potential study participant has been identified, they will be directed to the study website where they can complete a questionnaire to determine their eligibility.

Interested candidates will contact the Study Research Professional *via* phone or email (contact information supplied with recruitment materials) and will be asked to complete a general questionnaire online through the Research Electronic Data Capture (REDCap) system to determine eligibility. Information regarding participants' eligibility (whether eligible, ineligible, or withdrawn questionnaires), will be entered into the electronic Screening Log. Questionnaires that do not meet inclusion criteria will be discarded and the candidate will be informed of their ineligibility. Eligible questionnaires will be sent to study physicians for review. Candidates that meet eligibility will be contacted for a study overview session.

Following the study overview, informed consent will be obtained only by members of the research team who have been observed and approved by the Principal Investigator and authorized by the University of Arizona Institutional Review Board (IRB). Written informed consent will be obtained either online *via* REDCap or in person from each subject before the start of any study-related procedures and will be reviewed and signed and dated by the participant and the Study Research Professional in accordance with the Declaration of Helsinki. Ethical Approval for this study has been obtained from the University of Arizona IRB (Protocol #1200000220). We will recruit eligible participants who are able to provide informed consent and who are proficient or independent speakers of English or for whom English is their first language. As such, there is no provision or plan for obtaining consent from speakers of a language other than English.

### Screening Assessment

Participants who have provided informed consent, will undergo screening assessments in Visit 1 to determine whether they meet the enrollment criteria. Screening evaluations will be performed in sequence as follows:

- BP measurement: SBP ≥120 mmHg and <160 mmHg, DBP <100 mmHg after 5 min rest in seated position.
- Physical examination: assessments of the following parameters (jugular venous pressure, cervical lymph nodes, carotid pulse, normal breath sounds, regular heart rhythm, no abdominal swelling/tenderness, no swelling or discoloration of extremities, normal walking/gait, normal arm and leg strength, no rashes).
- Spirometry in accordance with guidelines from the American Thoracic Society (67): normal (FEV_1.0_) ≥80%.
- Height and weight measurement: BMI ≤ 40 kg/m^2^.
- Home sleep apnea test: Apnea Hypopnea Index ≥ 15 events/hour sleep (completed at home after Visit 1 and analyzed before Visit 2).
- Screening blood chemistries: Total cholesterol <240 mg/dL; Fasting plasma glucose <300 mg/dL.
- Epworth Sleepiness Scale score <15 (survey completed at home after Visit 1 but before Visit 2).

### Randomization, Blinding, and Treatment Allocation

Participants will be randomized after completing baseline assessments. Assignment to the training program will be by an unblinded Senior Research Professional. The study statistician will use a computer-generated randomization list for the study. Block randomization with block sizes of 2, 4, and 6 will be used to randomize participants into two groups (high- vs. low-resistance IMST) in a 1:1 ratio. Randomization will be stratified by sex (M/F) and OSA disease severity (moderate/severe). The randomization list will be stored in a .CSV file with the randomized group assignment hidden and only revealed by the Senior Research Professional at the time of randomization. Assignment to groups will be sequentially within strata.

Randomization will, on average, balance CPAP users (Yes/No) between the two arms within sex and severity strata however, the effect of treatment may differ depending on CPAP use. We will account for the potential effect of CPAP on outcomes, and possible interaction with treatment, by including CPAP and treatment-by-CPAP interaction effects in the mixed effects models. Based our pilot trials, we anticipate ~10–15% CPAP users in our study sample. The power to detect either a CPAP or a treatment-by-CPAP interaction, that is, our ability to tease out CPAP from IMST effects, will depend on the treatment-by-CPAP cell sample sizes and effect sizes.

Outcomes assessors and data analysts will be blinded to training group allocation. As both training programs have the potential to affect outcome measures, neither is considered a “placebo.” Subjects will be blinded to their allocation group.

### Study Withdrawal

Participants can withdraw consent at any time and for any reason if they wish to do so without harm of penalty. The researcher also may remove the participant from the study at any time if they believe study procedures have not been followed, for the benefit of the participant, or in the event of an adverse event (i.e., the development of severe side effects that, in the opinion of the supervising physicians, make it unsafe for the subject to continue in the study). Other discontinuation criteria will include cardiovascular or metabolic disease-related events (e.g., hypertensive crisis, myocardial ischemia/angina, myocardial infarction), major surgery, or other serious changes in physical or mental health status. The reason/s for withdrawal will be registered in the electronic Case Report Form.

### Adverse Events

An adverse event (AE) is any harmful and unintended reaction to a study assessment or intervention under investigation. Participants will be instructed to report side effects and AEs to members of the study team. All AEs occurring after the informed consent is signed and up until the subject has completed the study will be reported to the study physicians.

A serious adverse event (SAE) is any untoward medical occurrence that results in death, is life-threatening, requires inpatient hospitalization, results in persistent or significant disability/incapacity. In the event an SAE occurs, it will be reported to the University of Arizona IRB and the supervising physician within a maximum of 24 h from the moment the event is identified.

Throughout the study, the Principal Investigator will prepare yearly safety reports to the regulatory authorities and to the IRB at The University of Arizona following the schedule established in the current institutional guidelines.

### Statistical Analysis

The same statistical approach will be used to address all hypotheses, as each hypothesis uses data collected on continuous outcomes (e.g., casual and 24-h BP) from a two-arm design with multiple time points. A standard repeated measures (with the number of time points depending on outcome) mixed model analysis of variance will be used to estimate average change from baseline within arms. Models will adjust for stratification on OSA severity, sex, and age. Subject specific random effects will be used to account for the repeated measures on an individual. Inferences will be based on available data using maximum likelihood, which accommodates data missing at random. We propose a within-individual analysis for the study (comparisons between arms will be based on average change from baseline vs. average at endpoint) as the correlations between baseline and 6-week SBP and plasma norepinephrine in our preliminary data were ~0.60 or larger (49). Effect sizes and confidence intervals will be reported in addition to *p*-values. Unless otherwise mentioned, tests will be two-sided and conducted at the 5% significance level.

## Adherence

Successful adherence is defined as a study subject who achieves an adherence rate of at least 70% (i.e., completes at least 74 of the 106 training sessions and attends 10 of 14 in-person training sessions).

## Data Management

Data will be collected manually and or with case report forms (CRF) and source documents. CRFs are defined as an electronic document used to record all protocol relevant subject information. CRFs will generated and accessed through REDCap. CRFs are specific to each visit and will be completed for each subject. Research personnel responsible for visits will be required to complete the appropriate report form in an accurate and concise manner. Source documents are raw data documents where clinical observations are initially recorded. This includes laboratory reports, device reports, and physical examination reports. Information will be entered in CRF so that it follows what is included in source documents.

After the completion of a CRF, data will be exported into a master spreadsheet for analysis using a double-entry method. All data included in this sheet will be de-identified. Manually recorded data will be entered into a digital master data file within 1 week of data collection by the investigator who collected the measure. Data that has been acquired digitally (e.g., FMD_BA_, PWV_CF_) will be checked for completeness immediately after collection and then stored for later analysis. Data recorded at baseline, post-intervention, and follow-up for each subject will be analyzed individually after that subject completes all follow-up testing sessions to ensure within-subject accuracy.

## Data Monitoring

This study will have a Safety Officer who is completely independent of the sponsor. The Safety Officer is a physician-investigator with extensive experience in clinical trials on MA/O adults and adults with hypertension. The Safety Officer will meet with the Study Oversight Team at regular intervals (at least twice yearly), with one in-person meeting and the other *via* teleconference, to review enrollment progress, safety data, data quality, and the primary efficacy endpoints.

## Anticipated Results

### Primary Hypothesis

Twenty-four weeks of high-resistance IMST will lower casual SBP in MA/O adults with above-normal BP and OSA by an average of 15 mmHg; some BP reduction will persist after 4, but not 12 weeks of free living. Adherence to IMST will be high (>80% sessions).

### Other Hypotheses

Twenty-four weeks of high-resistance IMST will:

1. Lower 24-h, daytime, and nighttime SBP, as well as PNE.
2. Improve large elastic artery stiffness (i.e., PWV_CF_ reduction), NO-mediated EDD capacity, and *ex vivo* ROS production.
3. Reduce superoxide-related suppression of NO-mediated EDD (as assessed by FMD_BA_ with Vitamin C infusion).
4. Cause no change in EID (assessed as brachial artery dilation to sublingual nitroglycerin) indicating any improvements in FMD_BA_ observed are due to improvements in vascular endothelial function rather than increased vascular smooth muscle sensitivity to NO.
5. Lower casual DBP and 24-h, daytime, and nighttime DBP.

Some effects will persist after 4, but not 12 weeks of free living.

## Discussion

Brief-duration, high-resistance IMST is a simple, time-efficient breathing exercise that substantially lowers BP, and associated CVD risk, in a relatively short period of time (i.e., reductions of 6–12 mmHg after 6 weeks of daily training). These reductions have been reported in small groups of MA/O adults with above-normal BP (41), MA/O adults with OSA and above-normal BP (49), and normotensive young adults (50), but have not yet been established in a large-scale clinical trial. It is likely that performance of IMST over a longer time period (i.e., 24 weeks) will result in even greater reductions in BP, however, this has not been systematically assessed. The purpose of this study is to assess the effects of long-term, high-resistance IMST on BP in a large, representative group of adults with OSA. Additionally, we will assess the extent to which IMST-induced adaptations are sustained following training cessation. Finally, we will elucidate the underlying mechanisms that drive IMST-induced BP reductions by assessing the potential for high-resistance IMST to improve endothelial function, arterial stiffness, and vascular oxidative stress.

Adults with OSA are a unique clinical population because the prevalence of hypertension and CVD is higher than for the general population (3, 68). This is attributed to sleep fragmentation and nightly hypoxemic events that increase oxidative stress and sympathetic nervous system activity (69–71), and result in chronic increases in BP. The ability of IMST to reduce BP despite a chronic stimulus to increase BP is encouraging for other populations with above-normal BP. Further, because IMST is performed in a stationary sitting or standing position, it holds promise for many clinical populations that may be unable to perform traditional aerobic exercise and/or rehabilitation strategies (e.g., heart failure patients, mobility-limited older adults).

We do not expect any significant problems with these well-established training procedures that we have successfully employed previously (49, 52). In light of our previously published outcomes with IMST, our focus will be on outcomes for resting (casual) SBP (primary) and 24-h ambulatory SBP (secondary). FMD_BA_ and PWV_CF_ are strong independent predictors of incident CVD in MA/O adults (72, 73). Therefore, each of these vascular outcomes are important clinically as intermediary therapeutic targets of vascular disease. Measures of clinical CVD endpoints could be incorporated into a subsequent larger, more comprehensive, multi-site clinical trial. Although our ability to interrogate mechanisms of action in a definitive manner in all cases is limited, we have: (1) proposed mechanistic assessments based on findings from our foundational preclinical work in healthy young adults and (2) proposed to focus on assessments of NO-mediated vascular EDD because our pilot studies in MA/O adults without OSA support these as primary mechanisms (52). Last, recent prevalence estimates suggest >80% of adults >50 years exhibit age-related increases in aortic stiffness (elevated PWV_CF_) and/or hypertension (74) that is correlated with OSA severity (14). Thus, we anticipate a majority of subjects will exhibit aortic stiffening at baseline, and will benefit from IMST.

This study will assess the efficacy of long-term IMST in adults with OSA. Successful completion of this study will allow us to determine: (1) the extent to which IMST can lower BP over 24 weeks; (2) the degree of adherence to IMST when performed at home, 5 days/week, for 24 weeks; (3) persistence of training effects after cessation of IMST; and (4) the mechanisms driving IMST-induced reductions in BP, including effects on large elastic artery stiffness, oxidative stress-related suppression of NO-mediated EDD, and NO and ROS production. Ultimately, high-resistance IMST may prove to be an invaluable supplement to existing therapies for any population with above-normal blood pressure.

## Ethics and Dissemination

### Research Ethics Approval

This study has been approved by the University of Arizona Institutional Review Board (Approval Number: 1200000220).

### Protocol Amendments

Any modifications to the protocol which may impact the conduct of the study will first be agreed upon by the principal investigators and approved by the University of Arizona IRB prior to implementation. Administrative changes of the protocol are considered minor corrections that have no impact on the way the study is to be conducted. These changes will be agreed upon by the PI and documented.

### Consent

The study will be thoroughly explained to each subject over the phone, through an online video messaging platform, or in person, and subjects will have the opportunity to ask questions. Once the Senior Research Professional believes the subject understands the study requirements, they will be directed to an online platform to read and digitally sign the informed consent document. Additionally, there is a consent provision asking subjects if they are willing to allow remaining (unused) blood specimens to be used for future research studies. Subjects will have the option to accept or decline this provision without it affecting their participation status in the clinical trial.

### Confidentiality

Identity of subjects will be protected by assigning each a code (i.e., a 3-digit number), and any experimental data collected from these subjects will be recorded under that number. Any identifiable personal information will be kept in a password-protected digital file and/or in a locked cabinet. Only the PI, CO-I, Senior Research Professional, and Research Technician will have access to the information.

### Access to Data

The PI, CO-I, and Senior Research Professional will have access to the final trial dataset. Other project team members will be provided de-identified data for their analyses.

### Ancillary and Post-trial Care

There are no provisions for ancillary or post-trial care.

### Dissemination Policy

Primary outcome papers will be approved by the PI prior to journal submission. Every attempt will be made to release study results to the general public soon after study completion. Interim and final reports may also be presented at various local, regional, and international conferences, with approval from the PI. Eligibility for authorship include (1) substantial contribution to study conception and design AND/OR substantial contributions to acquisition, analysis, or interpretation of data, AND (2) drafting or revising the manuscript, AND (3) final approval of the manuscript. There is no intention to use professional writers.

## Ethics Statement

The studies involving human participants were reviewed and approved by University of Arizona Institutional Review Board. The patients/participants provided their written informed consent to participate in this study.

## Author Contributions

EFB, DS, and DC conceived of the study and initiated the study design. LR-B oversaw study coordination and implementation. DC provided technical training and oversight. JA and SM provided medical oversight. EJB provided statistical expertise. DT and EFB wrote the initial draft of the manuscript. All authors contributed to the refinement of the study protocol and approved the final manuscript.

## Funding

This work was supported by NIA/NIH Grant Number: 1R01AG065346-01A1 (EFB), NIH Training Grant Number: 5T32HL007249-44 (DT), and NIH/NHLBI Grant Number: 1K01HL153326-01 (DHC).

## Conflict of Interest

The authors declare that the research was conducted in the absence of any commercial or financial relationships that could be construed as a potential conflict of interest.

## Publisher's Note

All claims expressed in this article are solely those of the authors and do not necessarily represent those of their affiliated organizations, or those of the publisher, the editors and the reviewers. Any product that may be evaluated in this article, or claim that may be made by its manufacturer, is not guaranteed or endorsed by the publisher.
