# Supplementary material for: Six Months of Inspiratory Muscle Training to Lower Blood Pressure and Improve Endothelial Function in Middle-Aged and Older Adults With Above-Normal Blood Pressure and Obstructive Sleep Apnea: Protocol for the CHART Clinical Trial
Source: Front Cardiovasc Med. 2021 Nov 24;8:760203. doi: 10.3389/fcvm.2021.760203 (PMC8652071; doi:10.3389/fcvm.2021.760203)
Supplement: Supplementary file 1 [file Data_Sheet_1.docx]

Supplementary Material

# INTERNAL CONTROLS

**1.1 Anthropometrics**

1.1.1 Height

Subject height will be measured in centimeters with a flexible tape measure while the subject is standing flat footed, without shoes, against a wall.

1.1.2 Body Mass

Subject body mass will be measured in kilograms with a standard scale. The subject will be asked to remove their shoes, excess clothing (e.g., coat), and all objects in their pockets.

1.1.3 Neck circumference

Subject neck circumference will be measured in centimeters with a flexible tape measure around the thickest part of the neck while the subject is in a seated position.

**1.2 Surveys**

The following surveys will be completed at home in a single session between Visit 1 and Visit 2 at Baseline, after the 24-week intervention, and at 4- and 12-weeks after the intervention: Pittsburgh Sleep Quality Index (PSQI) [1], Epworth Sleepiness Scale (ESS) [1], Community Healthy Activities Model Program for Seniors (CHAMPS) physical activity questionnaire [2].

**1.3 Actigraphy**

Subjects will be provided a wGT3X-BT ActiGraph (ActiGraph, LLC, Pensacola, FL, USA) to be worn on the non-dominant wrist for 7 days to determine habitual physical activity level. Subjects will be instructed to wear the device continuously during the 7-day period. The Actigraph will be supplied to the subject at the first and last in-person exercise visits, to be returned after the 7-day period is complete.

**1.4 Spirometry**

Estimates of lung volume and airflow rates will be collected with the MiniSpir MIR spirometer (Medical International Research USA, Inc., New Berlin, WI, USA) and analyzed using Winspiro Pro software (Medical International Research USA, Inc., New Berlin, WI, USA) to determine if airflow limitations are present. Subjects will be fitted with nose plugs to prevent airflow through the nose. Subjects will then be instructed to inhale fully and then put the MiniSpir device in their mouth. They will then exhale as forcefully as possible until they can no longer exhale, at which point they will inhale as forcefully as possible until they can no longer inhale. Subjects will perform three trials with 1 minute of rest between trials.

**1.5 Sleep and Physical Activity Diary**

Subjects will record daily sleep characteristics (duration, sleep latency, waking events, wake time, and sleep quality) and physical activity characteristics (type, duration, and intensity) to ensure consistency of lifestyle behaviors.

**2 BLOOD PROCESSING**

Approximately 10 mL of blood will be collected after 20 minutes of supine rest during Visit 2 at baseline, post-intervention, and 4- and 12-week follow-up. Immediately after collection, the Research Technician will process the blood samples. The samples will be centrifuged at 1000 G-Force at 4º C for 20 minutes. Upon completion, plasma will be extracted with a 1000 µL pipette and separated into 1 mL cryotubes. Samples will be placed in -80º C freezer until batch analysis can be completed.

**3 REFERENCES**

1. Mondal P, Gjevre J, Taylor-Gjevre R, Lim H. Relationship between the Pittsburgh Sleep Quality Index and the Epworth Sleepiness Scale in a sleep laboratory referral population. Nat Sci Sleep. 2013;5:15–21. doi:10.2147/NSS.S40608

2. Hekler EB, Buman MP, Haskell WL, Conway TL, Cain KL, Sallis JF, et al. Reliability and validity of CHAMPS self-reported sedentary-to-vigorous intensity physical activity in older adults. J Phys Act Health. 2012;9(2):225–36. doi:10.1123/jpah.9.2.225
